# Poster Session I - A174 EXCEPTIONALLY RARE CASE OF RECTAL METASTASIS IN EARLY STAGE LUNG ADENOCARCINOMA

**DOI:** 10.1093/jcag/gwaf042.174

**Published:** 2026-02-13

**Authors:** N Chang, A Varghese, M Moini

**Affiliations:** Gastroenterology, McMaster University, Hamilton, ON, Canada; Health Sciences, McMaster University Faculty of Health Sciences, Hamilton, ON, Canada; Gastroenterology, McMaster University, Hamilton, ON, Canada

## Abstract

**Background:**

Lung cancer is the most common cause of cancer-related death worldwide and in Canada, with lung adenocarcinoma being the most common subtype. However, GI metastasis of lung adenocarcinoma is a rare phenomenon, with an incidence of 2.4%, and majority of which metastasizes most commonly to the small intestine, followed by the stomach and colon. Rectal metastases are exceedingly rare, with very few isolated reports available in the literature.

**Aims:**

To describe a patient with exceptionally rare rectal metastasis from stage IA lung adenocarcinoma, presenting with malignant ascites, persistent abdominal pain and diarrhea

**Methods:**

Case report and review of the literature

**Results:**

A 74 year old man underwent left upper lobectomy for stage IA lung adenocarcinoma in March 2025. Imaging and invasive staging (PET, EBUS of hilar/mediastinal nodes, and IR guided supraclavicular node biopsy) prior to the lobectomy showed no evidence of malignancy outside the left upper lobe. Meanwhile, patient had been having persistent diarrhea and weight loss since April 2024. Following the lobectomy, patient experienced progressively worsening symptoms, accompanied by abdominal distension and ascites. Cytological analysis of the ascitic fluid revealed malignant cells consistent with adenocarcinoma. A colonoscopy was performed and a rectal lesion was visualized, with biopsy demonstrating infiltrating adenocarcinoma in mucosa and superficial submucosa. Morphology and immunohistochemistry matched the prior pulmonary specimen, supporting metastatic lung adenocarcinoma rather than a new colorectal primary. Given marked functional decline and cachexia, systemic therapy was deferred in favor of palliation.

**Conclusions:**

High quality guidance for the detection and management of rectal metastases of primary lung adenocarcinoma remains limited. Presentation of metastases of the small and large intestine can be asymptomatic, but if there is a major complaint, it is generally abdominal pain; therefore, early recognition including consideration of colonoscopy could potentially create a window for intervention. Notably, in one other case of an isolated rectal metastasis of lung adenocarcinoma post lobectomy, it was detected early and surgically resected with a good early outcome. In contrast, our patient’s rapid decline and probable peritoneal spread limited therapeutic options and shifted the focus to comfort-oriented care. These contrasting clinical scenarios highlight the value of early evaluation of new gastrointestinal symptoms in the management of lung cancer, even early stage adenocarcinoma. While GI, and especially rectal, metastases remain a rare manifestation, prompt workup of new abdominal symptoms as part of lung cancer staging may identify the rare, treatable presentations and help improve individual outcomes.

**Funding Agencies:**

None

